# Supplementary material for: Chromothripsis during telomere crisis is independent of NHEJ, and consistent with a replicative origin
Source: Genome Res. 2019 May;29(5):737–49. doi: 10.1101/gr.240705.118 (PMC6499312; doi:10.1101/gr.240705.118)
Supplement: Supplemental Material [file supp_gr.240705.118_Supplemental_file_1.zip › contigs/annotated_contigs/DB45/contig.2.DB45_length_268_mean_cov_7.46268656716.docx]

**DB45_length_268_mean_cov_7.46268656716**

```
GTTATTGATCTCCCATTTTTATCACTAAAAATTGTGAAAACCCTTAGGTAGAGAAGCTACATAAATATTTCAGCCTCACCCTTTATCTT
 >chr18:70102324-70102440 - E=1e-58
ATCTTGTGATCCTCAAATCTTTTTT|TT|GTTATTTATATGCATCTGAGGATTGGAGGTGAGCCTTCAATACCTAATCCAGGATTCAAT
                          >chr18:70101392-70101546 - E=1e-81
TTAGAACATACAAATTATTCCACAAATTCTCACCATTTTCATAATTTTGAGACAGTCTTAAGATATTAAAAGATAAAGGCAAAAATTAC

TCT
```
